# The Modulatory Effect of tDCS Onset Timing in Alleviating Vigilance Decrement

**DOI:** 10.3390/brainsci15101085

**Published:** 2025-10-08

**Authors:** Zelin Pan, Yang Chen, Shanghong Wu, Tiansheng Xia

**Affiliations:** 1 School of Art and Design, Guangdong University of Technology, Guangzhou 510090, China; 12532863@mail.sustech.edu.cn (Z.P.); 3122007232@mail2.gdut.edu.cn (Y.C.); 3122007302@mail2.gdut.edu.cn (S.W.); 2 School of Automation and Intelligent Manufacturing, Southern University of Science and Technology, Shenzhen 518055, China

**Keywords:** tDCS, EEG, lower-frequency alpha, sustained attention, vigilance decrement, stimulus entry time

## Abstract

Vigilance refers to a sustained attentional state enabling the detection of specific but unpredictable changes in the external environment. This state typically declines rapidly over time. A deterioration in vigilance can lead to serious errors or accidents in both occupational and special scenarios, rendering vigilance intervention a critical area of interest for researchers. Transcranial direct current stimulation (tDCS) has shown promise in mitigating vigilance decrement. However, the timing of such interventions may yield differential effects, a question that remains unresolved in the literature. The present study examines the possibility of using the average power in the low alpha frequency band (alpha-1) as an Electroencephalography-based index of vigilance to identify a candidate entry point for tDCS application that may enhance efficacy, and further explores how the timing of tDCS influences vigilance outcomes. In the pilot experiment, we determined the timing for guiding tDCS based on the average power of the low alpha frequency band (alpha-1) from five participants, which was identified as the third stage of the experiment. The validity of this timing has been verified in subsequent independent samples with a larger size. In the formal experiment, ninety-nine participants were randomly assigned to three groups, receiving early intervention, late intervention, or a no-stimulus control, and completed a 20 min visual modification of the Bakan Task. The early-stimulated group (n = 33) received anodal stimulation (2 mA) on the right posterior parietal cortex during the first 8 min of the test (0–8 min), the late-stimulated group (n = 33) received stimulation on the same location during the middle 8 min of the test (8–16 min), while the blank control group (n = 33) received no stimulation. Results indicated that the late-stimulated group (8–16 min of stimulation), for which alpha-1 power guided the tDCS onset timing, was associated with a greater attenuation of vigilance decrement compared to the early-stimulated group (0–8 min of stimulation). Both groups demonstrated significant differences in vigilance during the first stage following stimulation.

## 1. Introduction

Vigilance is defined as a state of vigilance in which individuals are capable of detecting subtle, random, and predefined changes in the external environment and responding accordingly [1]. However, vigilance is not a single concept but rather encompasses distinct components that require differentiation [2,3]. When performing monotonous tasks that do not demand cognitive control—for instance, the Psychomotor Vigilance Task (PVT)—the arousal component of vigilance is primarily engaged. In contrast, tasks requiring cognitive control (e.g., the Sustained Attention Response Task, SART, which was utilized in the present study) involve the executive component of vigilance.

Maintaining such a high level of attentional focus over extended periods is inherently challenging and tends to deteriorate rapidly over time [4].In real-world contexts, this decrement can result in a range of hazardous consequences, particularly in critical occupational settings such as vehicular operation [4], aviation [5], and medical care [6]. Therefore, monitoring fluctuations in human vigilance and implementing timely interventions constitute essential safety strategies that have garnered considerable research attention.

A variety of methods have been developed for vigilance monitoring, with continuous psychophysiological monitoring recognized as one of the most effective for detecting vigilance loss. In previous studies and technological applications, researchers have employed heart rate variability [7], functional near-infrared spectroscopy [8], eye-tracking [9], electroencephalographic (EEG) signals [10], and multimodal physiological data [11] to detect and investigate vigilance loss. Among these, EEG-based monitoring methods have drawn particular interest due to their sensitivity to cognitive states. Various EEG markers of vigilance have been proposed and compared, with lower-frequency alpha power (alpha-1) emerging as one of the most reliable indices for detecting a decrement in vigilance during task performance. Decreases in vigilance have been found to negatively correlate with increases in alpha-1 power [12]. Further, studies utilizing tasks such as the Attentional Network Task–Interaction with Vigilance and Executive Attention (ANTI-Vea) have shown that alpha-band power increases over time as tasks progress [13,14], reinforcing the viability of alpha-1 power as a marker for monitoring vigilance levels. Additionally, earlier research [15] demonstrated the potential of using EEG-based engagement indices to monitor reading engagement in children. By triggering BCI-based training interventions when individualized engagement indices thresholds were met, children’s reading engagement was effectively enhanced. Such applications suggest a promising trajectory for EEG-triggered intervention strategies in vigilance monitoring.

To address rapid vigilance deterioration, researchers have explored diverse intervention strategies, ranging from everyday techniques (e.g., chewing gum [1], or consuming caffeinated products [16,17]) to neurostimulation methods like transcranial direct current stimulation (tDCS). By delivering non-invasive current to targeted brain regions, tDCS has been shown to improve vigilance across several experimental contexts [16,18,19,20]. Although outcomes vary depending on stimulation parameters, the overall body of evidence supports the potential of tDCS as a tool for cognitive enhancement in vigilance contexts. However, it is worth noting that existing studies are mostly based on acute tDCS interventions, and the long-term effects require further research [2,13]. Two recent studies [13,14] employing high-definition tDCS targeting the right posterior parietal cortex (rPPC) observed significant reductions in alpha power and concomitant improvements in vigilance scores relative to control conditions.

### The Current Study

Most existing studies on tDCS protocols designed to intervene in vigilance decrement have primarily focused on factors such as the location of brain stimulation, the duration of stimulation, or current intensity [21,22,23,24]. In contrast, far less attention has been devoted to the timing of stimulation during vigilance tasks. Although tDCS is known to produce long-lasting aftereffects [25], its immediate effects [26,27,28,29,30] are closely related to the stage at which the stimulation is delivered. At present, online stimulation has been shown to produce better outcomes than offline stimulation [31]. Furthermore, participants’ task performance has been found to depend on the precise timing of tDCS onset; the efficacy of tDCS has been attributed to its initiation effects [32], and the relative timing or delay between stimulation and task onset has been identified as a critical modulatory factor [33,34]. Collectively, these findings underscore a central principle: stimulation must be applied at the appropriate point in time—i.e., during the relevant task stage—to exert its intended effect [14]. Therefore, for online tDCS paradigms, establishing a reasonable entry point and duration for stimulation is crucial to maximizing its intervention efficacy.

Given that vigilance decrement is a gradual, progressive process, researchers commonly employ stage-based analyses to characterize and evaluate it [35,36]. Across different stages of vigilance decrement, the effectiveness of intervention strategies often varies. For example, studies have shown that rest breaks implemented during the early stages of vigilance decrement are far more effective than those introduced later. Moreover, as task time increases, the efficacy of brief rest periods in alleviating vigilance deterioration progressively diminishes [37]. Similarly, the mitigating effects of other intervention strategies also vary depending on the stage of vigilance decrement at which they are applied. Importantly, prolonged or continuous use of such interventions may result in adverse side effects or additional harm. For instance, excessive intake of stimulants like caffeine—commonly used to counteract vigilance loss—has been associated with increased risk of physical health complications [38,39,40]. Thus, identifying a potentially more effective stage for tDCS intervention during the vigilance decrement process at which interventions are most effective can enhance the overall efficacy of such methods while reducing unnecessary or ineffective usage. Technologies such as electroencephalography (EEG), heart rate variability, eye-tracking, and galvanic skin response are commonly employed for monitoring vigilance [41,42,43,44], among which EEG has been widely regarded as the “gold standard” for detecting changes in vigilance [44,45]. Compared to other vigilance monitoring techniques, EEG offers the distinct advantages of directly reflecting brain activity and being less susceptible to external disturbances [46]. Moreover, EEG signals exhibit nonlinear and highly dynamic characteristics [47]. In recent years, electroencephalography (EEG) monitoring has been extensively employed in the detection of vigilance decrement and in the investigation of objective standards for quantifying levels of vigilance [36,48]. Therefore, using a reliable EEG-based vigilance index to determine a putative tDCS entry point may enhance the stimulation’s potential mitigating effects on vigilance decrement. Accordingly, the primary aim of the present study is to use EEG-based vigilance indices to guide the timing of tDCS application and to examine whether the stage at which tDCS is introduced during the course of vigilance decrement modulates its effectiveness.

To this end, we selected the power of the alpha-1 frequency band—a metric previously demonstrated to be highly sensitive to changes in vigilance [12,42]—as the EEG index for guiding the timing of tDCS intervention in this study. Regarding stimulation sites, we adopted the same electrode placements as in prior research [13,14] to apply tDCS to the right posterior parietal cortex (PPC) and monitor EEG signals in the right PPC and right dorsolateral prefrontal cortex (DLPFC), both of which are considered core components of the attentional control network [49,50]. High-definition transcranial direct current stimulation (HD-tDCS) was used as the stimulation protocol. For the vigilance assessment, we designed a modified task based on the Bakan vigilance test [51], which enables the observation of vigilance decrement over a relatively short time span. Concerning the differences in tDCS onset timing, the present study focused on variations in the specific task stage during which stimulation was initiated and sustained within the overall vigilance task cycle. We implemented a between-subjects experimental design in which the only difference between groups was whether the timing of tDCS onset was determined based on the alpha-1 index.

## 2. Materials and Methods

### 2.1. Participants

A total of 117 students (58 females and 59 males) from Guangdong University of Technology voluntarily participated in this study. The participants had a mean age of 20.56 years (SD = 1.48). Most participants were college-educated students, and the numbers were balanced with respect to gender. All participants were right-handed, had normal or corrected-to-normal vision, and were not color blind or color deficient. Demographic information for the study cohort is presented in Table 1. All individuals expressing interest in this study must complete a telephone screening with the research coordinator prior to the experiment. Eligibility criteria stipulate that participants must: (1) be available for daytime appointments outside of rest periods; (2) agree to abstain from alcohol and stay awake the night before the study visit; (3) be free of physical trauma and self-identify as generally healthy; (4) have normal vision or vision corrected to normal. Additionally, individuals are ineligible if they: (1) have significant medical or neurological conditions that could interfere with study results; (2) regularly take medications or substances known to impair study results or EEG recordings (e.g., benzodiazepines); (3) report frequent insomnia and poor sleep hygiene; (4) show skin damage at stimulation sites; or (5) suffer from skin conditions such as dermatitis, psoriasis, or eczema will be excluded from volunteering. Participants were randomly assigned to the early-stimulated group (n = 38), the late-stimulated group (n = 37), and the blank control group (n = 42). A pre-hoc power analysis was conducted using G*Power 3.1.9.7 for the three-group (early/late/no stimulus × 5-stage mixed design): Effect size f = 0.25 [14], Cohen’s d = 0.80, α = 0.05, N = 96, 1 − β ≈ 0.80, the total sample size required > 96 participants (more than 32 per group), thus meeting statistical standards. The study protocol was approved by the Academic Ethics Committee of Guangdong University of Technology (No. GDUTXS20250131).

### 2.2. Vigilance Task: Visualized Modified Version of the Bakan Task

The vigilance task was developed using Python 3.13 and was based on a visually modified version of the original Bakan Task [51], taking reference from previously validated short-form vigilance paradigms [52]. The task lasted for a total of 21 min, with the first minute serving as an adjustment period and excluded from analysis. The remaining 20 min were divided into five testing stages of 4 min each.

**Figure 1:**
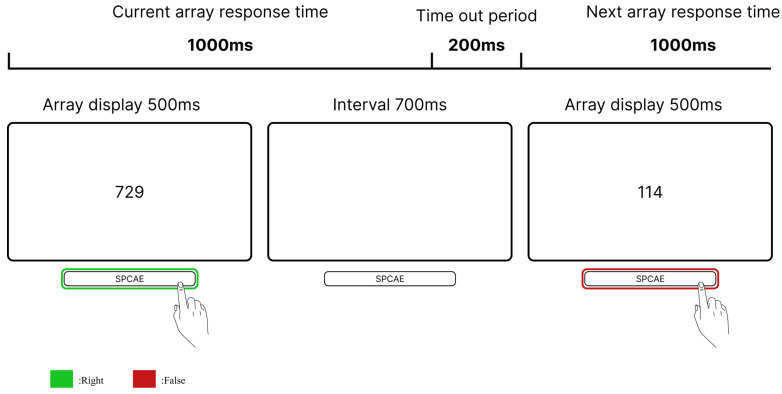
Visualized modified version of the Bakan Task.

In each minute, 50 sequences of digits were presented. Each sequence consisted of three randomly selected digits ranging from 1 to 9. Each triplet appeared on screen for 500 ms and was followed by a 700 ms inter-stimulus interval. Target sequences conformed to an “odd-even-odd” pattern, such as “729” (see Figure 1). Participants are instructed to press the spacebar as quickly as possible when the target array appears. The criterion for hitting the target was a reaction time less than the adaptive threshold time (individual average reaction time + 2 standard deviations), while a miss was recorded if the reaction time exceeded the adaptive threshold. Reactions to non-target arrays are recorded as false alarms. Target sequences appeared with a frequency of 20% per minute. To minimize practice effects during the formal session, participants completed a 4 min initial practice followed by an 8 min extended practice before beginning the main task.

The task was displayed on a 1920 × 1080 pixel screen with a white background. All number sequences were centrally presented in Inter font at a size of 150. No performance feedback or cues regarding correctness were provided throughout the task.

### 2.3. Subjective Mood Measurement

Before any experimental procedures, participants completed the Visual Analogue Mood Scale [53] to assess their subjective emotional state. The scale adopts a 5-point Likert format and includes 16 items (see Table 2) covering three independent affective dimensions: vigilance, contentment, and calmness. Participants rated each item to indicate their current emotional status. The same scale was administered again at the end of the experiment to assess changes in subjective mood following the intervention.

### 2.4. Stimulation Protocols and EEG Recording

#### 2.4.1. Apparatus

The tDCS protocol and EEG signal acquisition were conducted using the Starstim^®^ system, an eight-channel wireless neurostimulation and EEG recording device, and were controlled via NIC software version 2.1.3.7 (Neuroelectrics^®^, Barcelona, Spain). Eight hybrid NG Pistim electrodes (with a 12 mm Ag/AgCl sintered pellet and a circular contact area of 3.14 cm^2^) were embedded in a neoprene EEG cap aligned with the international 10-10 EEG system over 39 electrode positions. A reference EarClip electrode was affixed to the participant’s right earlobe. tDCS was delivered using a 4 × 1 ring configuration, consisting of a central anode surrounded by four return cathodes arranged in a circular array [54].

#### 2.4.2. tDCS Setup and EEG Recordings

**Figure 2:**
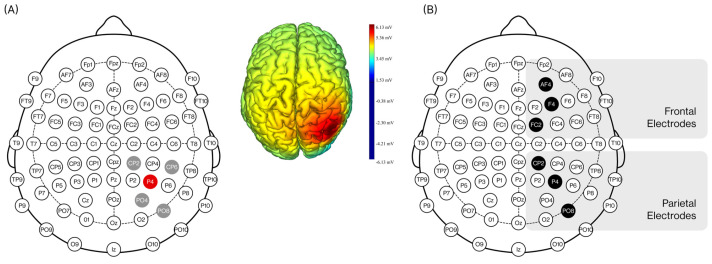
(**A**) Illustration of the 4 × 1 anodal tDCS setup: the anode is placed over P4 (red electrode), with return currents flowing through CP2, CP6, PO4, and PO8 (gray electrodes), producing the simulated voltage field shown on the right; (**B**) EEG signals were recorded before and after the stimulation period using six electrodes (black electrodes), with parietal regions at CP2, P4, and PO8, and frontal regions at AF4, F4, and FC2.

The eight electrodes were distributed across the right frontal and right parietal cortical regions. The anodal electrode was positioned at P4, while the four return (cathodal) electrodes were placed at CP2, CP6, PO4, and PO8. During the stimulation period, anodal tDCS (2 mA) was administered over the right posterior parietal cortex (PPC), and EEG data were recorded from both right frontal and right parietal regions during non-stimulation periods. For both the experimental and control conditions, each stimulation stage included two ramping periods: 30 s for ramp-up and 30 s for ramp-down. Figure 2A illustrates the electrode setup of tDCS and the resulting simulated voltage field (extracted from the NIC software, Neuroelectrics^®^).

EEG recordings were acquired from six electrodes, as shown in Figure 2B: AF4, F4, and FC2 (right frontal region), and CP2, P4, and PO8 (right parietal region). EEG signals were recorded both before and after the stimulation sessions. The sampling rate was 500 Hz, with a recording bandwidth of 0–125 Hz and a 50 Hz notch filter applied to eliminate power-line noise.

All EEG data were preprocessed using EEGLAB in MATLAB R2024a. A high-pass filter at 0.5 Hz and a low-pass filter at 30 Hz were applied. Independent Component Analysis (ICA) was conducted to identify and remove artifacts.

### 2.5. Procedures and Pilot Study

#### 2.5.1. Pilot Study and Its Results

**Figure 3:**
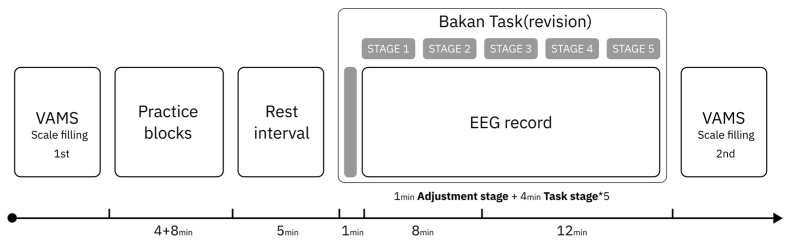
Pilot study flowchart.

**Figure 4:**
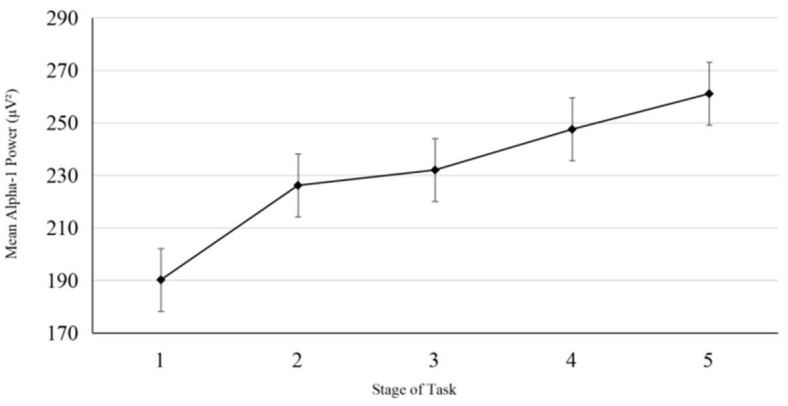
Mean alpha-1 power per task stage in the pilot (error bars are standard errors of the Mean).

In order to explore potential timing for tDCS intervention guided by the alpha-1 power index, a pilot study was conducted prior to the formal experiment. Five participants (three females and two males; M age = 20.2 years, SD = 0.447) completed the vigilance task identical in all aspects to the main experiment, including full EEG signal recording throughout the task. No tDCS was applied during the pilot study. The experimental procedure (see Figure 3) mirrored that of the formal experiment. As in the main study, the 20 min task duration was divided into five consecutive 4 min stages. In the pilot experiment, we observed that participants’ alpha-1 power typically reached the mean level calculated across the entire duration of the task (comprising five stages over 20 min) around the third stage. (see Figure 4) We interpreted this as an indication that participants had entered a stage of stable vigilance decrement. Accordingly, we designated the third stage as a putative stimulation onset time point for tDCS intervention guided by the alpha-1 index, which served as a group-level reference for the formal experiment.

#### 2.5.2. Experimental Procedures

**Figure 5:**
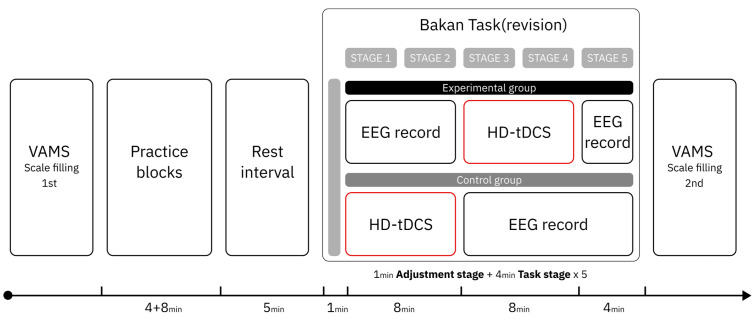
Experiment flowchart.

Prior to the start of the experiment, all participants completed the first administration of the Visual Analogue Mood Scale (VAMS) scale and received a standardized explanation of the vigilance task from the experimenter. According to the results of the pilot study, participants in the late-stimulated group received transcranial direct current stimulation at a current intensity of 2 mA and a total duration of 8 min—comprising a 30 s ramp-up and a 30 s ramp-down, with 7 min of effective stimulation—beginning in stage 3 of the formal vigilance task, which was determined as the stimulation onset based on the alpha-1 power index. In contrast, the early-stimulated group did not follow this alpha-1-guided timing protocol; instead, they received the same tDCS protocol (identical in intensity and duration) starting in stage 1 of the formal task. The blank control group did not receive any stimulation and completed all five stages of the task. Aside from the difference in stimulation onset timing, the experimental procedure (see Figure 5) was identical between the three groups. Upon completing the task, all participants completed VAMS for the second time.

### 2.6. Statistical Analyses

#### 2.6.1. Behavioral Data

The vigilance task score is calculated using d’ [55]. The d’ is a classic indicator used in Signal Detection Theory (SDT) to measure perceptual sensitivity and serves as an objective measure of participants’ vigilance in this study. The formula is

d’ = z (Hit Rate) − z (False Alarm Rate)

Hit Rate: The proportion of correctly identifying signals when they are present. False Alarm Rate: The proportion of incorrectly reporting signals as present when they are absent. z(x): Represents the z-value of the standard normal distribution corresponding to x, i.e., the inverse of the cumulative distribution function.

In this study, the d’ values across five stages were used as an objective criterion to assess participants’ vigilance. Eighteen participants (8 males, 10 females) were excluded due to technical connection issues during data collection. Specifically, 5 participants (2 males, 3 females) were excluded from the early-stimulated group, 4 participants (2 males, 2 females) from the late-stimulated group, and 9 participants (4 males, 5 females) from the blank control group. Ultimately, the early-stimulated group comprised 33 participants (17 males, 16 females), the late-stimulated group comprised 33 participants (16 males, 17 females), and the blank control group comprised 33 participants (18 males, 15 females), with a total of 99 participants. This still met the required total sample size of >96 participants (≥32 per group) for power analysis, thus adhering to statistical norms. In our analysis, we excluded the first minute of the adjustment stage and calculated d′ for each of the five 4 min stages over the total 20 min period. The stage of the vigilance task was treated as a within-subject factor, and group (Blank control group vs. Early-stimulated group vs. Late-stimulated group) was treated as a between-subject factor. A Mixed-design analysis of variance (ANOVA) was conducted to examine changes in d′ across stages and between groups. Meanwhile, to gain a broader understanding of the impact of tDCS on vigilance performance, we also conducted a mixed-design analysis of variance (ANOVA) on the hit rates, false alarm rates, and reaction time across all stages for each group to observe the evolution of different behavioral metrics.

#### 2.6.2. EEG Data

EEG signal detection was performed in all stages for the blank control group. For the early-stimulated group, EEG signal detection was performed in stages 3, 4, and 5, while for the late-stimulated group, it was performed in stages 1, 2, and 5. We first validated the key conclusion—the behavioral-EEG correlation—based on the EEG data and d’ of vigilance performance from the five stages of the blank control group: the negative correlation between alpha-1 power and vigilance performance. To better compare the effects of stimulation on EEG data across different time periods, we decided to use the blank group as the standard baseline for comparison, i.e., comparing the difference between the average alpha-1 power of the blank control group and the early-stimulated group in stage 3 with the difference between the average alpha-1 power of the blank control group and the late-stimulated group in stage 5.

In the EEG data analysis stage, the final data was obtained from 99 participants (33 in the early-stimulated group, 33 in the late-stimulated group, and 33 in the blank control group) from six channels in the frontal lobe region (AF4, F4, and FC2) and the right parietal lobe (CP2, P4, and PO8) were used as basic data for analysis. An independent samples *t*-test was performed on the difference in alpha-1 average power between the two stages.

## 3. Results

### 3.1. Pre-Verification

Since our experiment was based on a key conclusion, the behavioral-EEG correlation: the negative correlation between alpha-1 power and vigilance performance, we validated the tDCS onset timing guided by the average power of the low alpha frequency band (alpha-1) in the pilot study using a larger sample size. Therefore, based on the collected EEG data, we began with a discussion.

**Figure 6:**
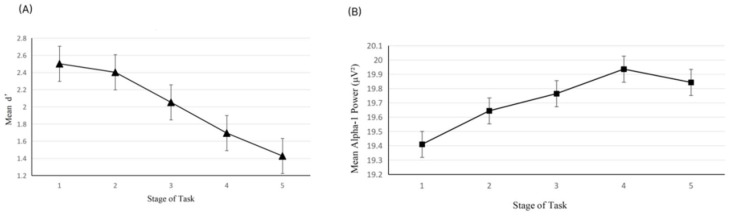
(**A**) Mean d’ per task block in the Blank Control Group (error bars are standard errors of the Mean); (**B**) Mean alpha-1 power per task block in the Blank Control Group (error bars are standard errors of the Mean).

We found that participants’ alpha-1 power generally reached the overall alpha-1 power average (across 5 stages totaling 20 min) around stage 3 (see Figure 6B). These results from the blank control group (n = 35) replicated the key findings from the pilot study: stage 3 is the critical turning point when vigilance enters a stable decline phase (as indicated by alpha-1 power). This cross-validation based on a larger sample reduces the risk of over-generalizing conclusions from the small sample pilot study and supports the rationale for selecting stage 3 as the stimulation onset time.

We conducted a repeated-measures ANOVA on the alpha-1 power of the blank control group across five stages. The results showed that the Mauchly sphericity test indicated that the sphericity assumption was not met (*p* < 0.001), and the Greenhouse-Geisser correction was applied. The main effect of stage was significant, F (4, 128) = 3.523, *p* = 0.027, $\eta_{p}^{2}$ = 0.094. Post hoc comparisons revealed a marginally significant linear trend, F (1, 34) = 7.853, *p* = 0.080, indicating an overall increase in alpha-1 power across stages; the quadratic trend was marginally significant, *p* = 0.050. Paired comparisons (Bonferroni corrected) did not reveal any significant direct differences between any two stages (*p* > 0.05), but the graph shows that alpha-1 power increased as the experimental task progressed (see Figure 6A). For the blank control group, to address the repeated-measures structure (five task stages per participant), we examined the relationship between alpha-1 power and vigilance score (d’) using a Linear Mixed Model (LMM). The LMM specified d’ as the dependent variable, alpha-1 power as the key predictor, task stage as a fixed effect (to control temporal fluctuations), and Subject ID as a random intercept (to account for inter-individual differences) [56]. Results showed a significant negative association between alpha-1 power and d’ (β = −0.048, SE = 0.024, t = −2.041, *p* = 0.045, 95% CI [−0.096, −0.001]), confirming that higher alpha-1 power was linked to lower vigilance (see Figure 6B).

### 3.2. Behavioral Level Analysis

The vigilance task divided a 20 min task into five stages and calculated the d’ value for each stage.

**Figure 7:**
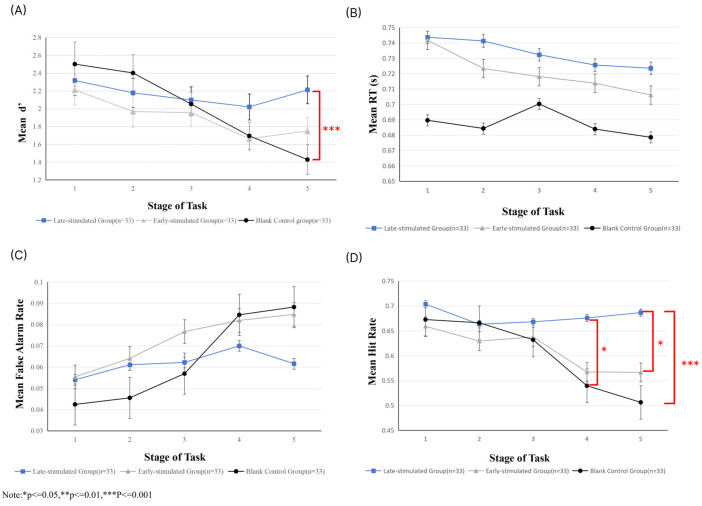
(**A**) Mean d′ per stage (error bars are standard errors of the Mean) (**B**) RT Average (s) per stage (error bars are standard errors of the Mean) (**C**) False Alarm Rate Average per stage (error bars are standard errors of the Mean) (**D**) Hit Rate Average per stage (error bars are standard errors of the Mean).

Subsequently, for d’, we conducted a Mixed-design ANOVA. The analysis showed that the main effect of group was not significant (F (2, 96) = 1.064, *p* = 0.349, $\eta_{p}^{2}$ = 0.022), indicating no overall difference in vigilance across the three groups. However, the main effect of stage was significant (F (4, 93) = 9.487, *p* < 0.001, $\eta_{p}^{2}$ = 0.290), suggesting a decrease in vigilance over time. The interaction effect between group and stage was also significant (F (8, 188) = 4.172, *p* < 0.001, $\eta_{p}^{2}$ = 0.151), showing that the groups differed in how their vigilance declined. Additionally, the simple effects analysis (Bonferroni-corrected) revealed no significant between-group differences in vigilance during stages 1–4 (*p* > 0.05), suggesting that the experimental treatment did not noticeably affect vigilance during either the stimulus implementation or the immediate post-stimulation stage. However, in stage 5 (immediately after the late-stimulated group’s stimulus), significant differences in vigilance were observed between the late-stimulated and blank control groups (*p* < 0.01). No significant difference was found between the early-stimulated and blank control groups (*p* > 0.05). Therefore, these results suggest that: that no differences were observed in stage 1, indicating that the initial baseline vigilance levels of the three groups were similar, ruling out group-related biases; no differences were observed in stage 3 (the immediate stage after the early-stimulated group’s stimulus), indicating that the early-stimulus group did not alter the natural decline in vigilance over time; however, the intergroup differences in stage 5 indicate that the late-stimulus group exerted a regulatory effect on vigilance, significantly mitigated its decline (see Figure 7A).

Reaction time (RT) data were analyzed using repeated-measures analysis of variance (ANOVA). The results revealed a significant main effect of stage, F (4, 384) = 6.628, *p* < 0.001, $\eta_{p}^{2}$ = 0.065. However, the trend in RT was decreasing. Based on this finding, the visually modified Bakan Task employed in the present study can be classified as a repetitive cognitive discrimination task. In such tasks, participants typically exhibit a practice effect [57]: as the task progresses, their ability to anticipate stimulus sequences and their proficiency in motor responses improve. This practice effect may counteract, or even outweigh, the tendency for RTs to lengthen due to vigilance decrement. A significant main effect of group was also observed, F (2, 96) = 3.833, *p* = 0.025, $\eta_{p}^{2}$ = 0.074, indicating that overall RTs differed across stimulus conditions. Moreover, a significant stage x group interaction emerged, F (8, 384) = 2.403, *p* = 0.015, $\eta_{p}^{2}$ = 0.048. However, due to the confounding influence of practice effects, this interaction will not be discussed further (see Figure 7B).

With respect to false alarm rate, the main effect of stage was significant, F (4, 384) = 22.648, *p* < 0.001, $\eta_{p}^{2}$ = 0.191, showing an overall increasing trend in false alarm rate, which is consistent with the classical cognitive pattern of vigilance decrement (see Figure 7C). The main effect of group was not significant, F (2, 96) = 0.865, *p* = 0.424, $\eta_{p}^{2}$ = 0.018, suggesting no reliable differences in overall false alarm rate among the late-stimulated group, the early-stimulated group, and the blank control group. This indicates that transcranial direct current stimulation (tDCS) did not exert a consistent effect on “average false alarm rate across all stages.” However, the stage x group interaction was significant, F (8, 384) = 4.561, *p* < 0.01, $\eta_{p}^{2}$ = 0.087. None of the simple effects reached statistical significance. Nevertheless, visual inspection of the data (see Figure 7C) suggests that the blank control group exhibited a steeper increase in false alarm rate during the later stages (Stages 4 and 5), whereas the late-stimulated group showed a relative reduction in false alarm rate at Stage 5. These trends did not reach significance, likely due to limited statistical power. Future studies could address this issue by increasing sample size or optimizing stimulation paradigms to more clearly elucidate stage-specific effects of tDCS on false alarm rate.

For hit rate, the main effect of stage was significant, F (4, 384) = 12.309, *p* < 0.001, $\eta_{p}^{2}$ = 0.114, indicating systematic changes across stages. The main effect of group was not significant, F (2, 96) = 2.135, *p* = 0.124, $\eta_{p}^{2}$ = 0.043, suggesting no reliable differences in average hit rates across the three groups. However, the stage x group interaction was significant, F (8, 384) = 4.384, *p* < 0.01, $\eta_{p}^{2}$ = 0.084. Further simple-effect analyses (Bonferroni-corrected) showed that, when comparing groups within each stage, no significant differences were observed among the three groups during Stages 1–3 (*p* > 0.05). This indicates that during the early phases of the task, regardless of whether participants received tDCS or the stimulation timing, their hit rate performance remained comparable, and the effect of the intervention had not yet emerged. At stage 4, however, the hit rate of the late-stimulated group was significantly higher than that of the blank control group (MD = 0.143, *p* = 0.011), whereas no significant difference was observed between the late-stimulated and early-stimulated groups (*p* = 1.000). This suggests that when the task reached the mid-stage—corresponding to the critical window in which vigilance begins to decline steadily, as indexed by the alpha-1 power marker—late stimulation was able to maintain a superior level of hit rate, while early stimulation did not yet show a significant difference. By stage 5, the hit rate of the late-stimulated group was significantly higher than both the early-stimulated group (MD = 0.123, *p* = 0.024) and the blank control group (MD = 0.202, *p* < 0.001). As shown in Figure 7D, both the blank control group and the early-stimulated group exhibited a marked decline in hit rate toward the end of the task, whereas the late-stimulated group was able to maintain a relatively high level. This result strongly supports the study hypothesis—that “late tDCS,” guided by alpha-1 power as an EEG marker, can be precisely aligned with the dynamic process of vigilance decrement, thereby effectively sustaining hit rate performance during the later stages of the task.

### 3.3. EEG Data Analysis

To assess whether there were significant differences in the average alpha-1 power among the three groups after stimulation, first, independent *t*-tests (Bonferroni-corrected) were conducted between the alpha-1 power of the blank control group at Stages 3 and 5 and the alpha-1 power of the early-stimulated group at Stage 3 and the late-stimulated group at Stage 5. To exclude the influence of task duration on alpha-1 power, independent t-tests were then performed to compare the difference in alpha-1 power between the blank control group and the early-stimulated group at stage 3 with the difference in alpha-1 power between the blank control group and the late-stimulated group at stage 5.

The results (see Table 3) showed that alpha-1 power decreased at stage 3, but this trend was not significant. However, the decrease at stage 5 was significant. The difference in alpha-1 power between stage 3 (M = 0.85, SD = 3.55) and stage 5 (M = 2.01, SD = 3.66) was significant (t (64) = 2.17, *p* = 0.034, Cohen’s d = 0.533), indicating a moderate effect. Therefore, based on this comparison of differences, it can be observed that alpha-1 power decreases regardless of the stage at which stimulation is applied; however, the decrease is more pronounced and statistically significant in stage 5 following stimulation in stage 3; Secondly, the comparison of differences indicates that stimulation in stages 3 and 4 has a greater impact on alpha-1 power than stimulation in stages 1 and 2. Based on the negative correlation between changes in alpha-1 power and changes in vigilance, this suggests better vigilance intervention effects in the late-stimulated group at the alpha-1 power level.

### 3.4. VAMS Score

Across all three VAMS dimensions, the covariates were unrelated to the experimental manipulation and satisfied the assumption of homogeneity of regression slopes. We used a mixed-design ANOVA to analyze the three VAMS dimensions (vigilance, contentedness, and calmness), with “group” as the between-subject factor and “pre-experiment vs. post-experiment” as the within-subject factor.

Vigilance dimension: The main effect was not significant before and after the experiment, F (1, 96) = 0.073, *p* = 0.788, $\eta_{p}^{2}$ = 0.001; The main effect of group was not significant, F (1, 96) = 1.609, *p* = 0.205, $\eta_{p}^{2}$ = 0.032; The interaction effect between group and stage was not significant, F (2, 96) = 1.483, *p* = 0.232, $\eta_{p}^{2}$ = 0.030.

Satisfaction dimension: The main effect was not significant before and after the experiment, F (1, 96) = 3.381, *p* = 0.069, $\eta_{p}^{2}$ = 0.034; The main effect of group was not significant, F (2, 96) = 1.910, *p* = 0.154, $\eta_{p}^{2}$ = 0.038; The interaction effect between group and stage was not significant, F (2, 96) = 2.476, *p* = 0.089, $\eta_{p}^{2}$ = 0.049.

Calmness dimension: The main effect was not significant before and after the experiment, F (1, 96) = 2.408, *p* = 0.124, $\eta_{p}^{2}$ = 0.024; The main effect of group was not significant, F (2, 96) = 2.909, *p* = 0.059, $\eta_{p}^{2}$ = 0.057; The interaction effect between group and stage was not significant, F (2, 96) = 3.337, *p* = 0.060, $\eta_{p}^{2}$ = 0.045.

Although the three groups showed some changes in the dimensions of vigilance, satisfaction, and calmness on the VAMS, the main effect of group did not reach statistical significance. This result can be explained from two core aspects: first, subjective reports have inherent limitations—their processing of neurocognitive changes is not only delayed but also significantly less sensitive than objective indicators such as EEG and task performance. As previous studies have shown, fatigue states that cannot be captured by self-reports or reaction times can be directly detected using EEG and functional near-infrared spectroscopy [58]. Secondly, further confirmed in sleep deprivation studies that subjective alertness, objective cognitive performance, and brain electrical activity are regulated by different neural mechanisms [59]. This dissociation between subjective and objective measures is stable within individuals, and subjective reports consistently lag behind objective indicators in capturing changes in neural function.

## 4. Discussion

This study aimed to investigate the efficacy enhancement of tDCS on mitigating vigilance decrement by adjusting the stimulation onset based on EEG-based vigilance indicators. To this end, we employed the well-validated metric of alpha-1 band mean power as the EEG marker of vigilance, as supported by previous studies [60,61,62,63,64]. Regarding the vigilance task, we adapted the Bakan paradigm by incorporating the simplified structure of the Temple vigilance task [52,65,66] to suit the specific goals of the current study. Our primary focus was on the direct impact of differences in tDCS timing on overall vigilance performance. Therefore, the study employed a simplified vigilance task paradigm, rather than the attentional network tests utilized in some other investigations [13,14,67,68,69]. Through a pilot study, we identified the temporal window during which participants’ alpha-1 power reached its average level during the vigilance task. This window was then used to define group-level differences in stimulation onset in the main experiment. Given that the focus of our research was to examine the differential effects of stimulation timing rather than the presence versus absence of tDCS, in the formal experiment, we divided the participants into three groups. Participants in the blank control group underwent continuous EEG signal monitoring to verify the negative correlation between alpha-1 average power and vigilance performance. Participants in the early-stimulated and late-stimulated groups received tDCS with identical settings, with the only difference being the timing of tDCS administration. The late-stimulated group began stimulation during the time period when alpha-1 reached its mean value (8–16 min), while the early-stimulated group received stimulation during the initial stage of the task (0–8 min).

In the dimension of vigilance, mixed-factor analysis revealed that vigilance significantly declined over time in all three groups, with significant differences in the trend of vigilance decline between the groups. Simple effects analysis showed that the initial vigilance baseline was consistent across the three groups, eliminating any group-related bias. Early stimulation did not alter the natural decline in vigilance over time, while late stimulation was associated with a weaker vigilance decrement trajectory, which may reflect a modulatory role in vigilance maintenance. The most notable differences in the experiment were observed in the changes in vigilance scores and EEG data between stages 3 and 5. Within the 20 min task duration, the two groups exhibited significant differences following stimulation. Both d’ values and alpha-1 average power reflected the participants’ objective vigilance performance. The early-stimulated group received intervention at a stage of lower vigilance, while the late-stimulated group was intervened at a higher vigilance stage. The results indicated that the late-stimulated group showed significantly better vigilance task performance than the early-stimulated group. This suggests that early tDCS intervention did not significantly improve vigilance and exhibited delayed effects. In the first stage after stimulation, the late-stimulated group outperformed the early-stimulated group in both vigilance scores and alpha-1 power, indicating that tDCS intervention during the middle stage of vigilance decline is more effective in alleviating the decrement. These results contrast with findings from some studies [37] suggesting that early brief breaks are more beneficial, potentially due to differences in task duration. In shorter tasks, tDCS intervention during the vigilance decline phase, as reflected by alpha-1 power, showed greater effectiveness compared to early intervention. This highlights the crucial role of the timing of tDCS in modulating vigilance decline.

In the EEG data analysis, repeated measures ANOVA on the blank control group’s alpha-1 power across five stages showed an upward trend, with a negative correlation between alpha-1 power and vigilance performance (d’ scores). Independent t-tests compared alpha-1 power between the blank control group (stages 3 and 5), the early-stimulated group (stage 3), and the late-stimulated group (stage 5). To exclude the influence of task duration on αlpha-1 power, we used an independent samples t-test to analyze the differences in αlpha-1 power between the blank control group and the early stimulation group in the third stage, as well as between the blank control group and the late stimulation group in the fifth stage.

The results showed that (1) alpha-1 power decreased in all stages compared to the blank control group, and (2) the late-stimulated group had a better vigilance intervention effect, as reflected by alpha-1 power, due to its negative correlation with vigilance decline.

An increasing number of studies are now exploring the modulatory effects of tDCS on vigilance. In terms of tDCS protocols, the core research focus tends to be on the stimulated brain regions and the differences in outcomes associated with these regions [13,20,69]. However, beyond stimulation site and duration, the temporal stage at which stimulation is applied is also a critical determinant of tDCS efficacy. Previous studies typically aligned the duration of online tDCS with the full length of the vigilance task [13], thereby covering the entire testing period. This approach prevents the evaluation of potential differences in stimulation efficacy across different stages of vigilance decrement. In the present study, the duration of tDCS administration during the task was deliberately shortened, allowing the stimulation period to fall within different stages of the vigilance decrement trajectory across experimental groups. This design enabled a direct comparison of stimulation efficacy at distinct stages of vigilance deterioration.

Using alpha-1 mean power as a marker of vigilance deterioration is a widely accepted method, and our findings further reinforce its validity in reflecting changes in vigilance. The lower alpha-1 power observed in the late-stimulated group during the final stage may reflect a more alert cognitive state. The efficacy of tDCS is known to be influenced by individual differences [70,71]. Due to practical constraints, our study did not compute personalized stimulation timings for each participant based on individual alpha-1 trends. Instead, we adopted a generalized timing window derived from pilot data. Despite this limitation, the late-stimulated group—whose stimulation timing was guided by the alpha-1 mean—still demonstrated superior performance. Future studies might benefit from tailoring stimulation onset to individualized alpha-1 dynamics, potentially yielding even more pronounced effects. Previous work has demonstrated the utility of personalized EEG-guided intervention strategies [15].

Subjective assessments of vigilance-related affective states did not reveal statistically significant group differences. Correlational analyses indicated that changes in subjective vigilance ratings and objective task performance may reflect distinct vigilance mechanisms. Indeed, several studies also suggest that the correlation between physiological indicators and subjective ratings is relatively weak [72]. In terms of the subjective perception of vigilance, no differences were observed in the effects of tDCS applied at different stages.

According to the multi-component theory of vigilance [2,3], vigilance is divided into different components, such as executive vigilance (EV) and arousal vigilance (AV). When tasks require maintaining vigilance to respond to rapid changes and rare events, executive vigilance becomes particularly important. The task used in our study is a relatively complex one, requiring participants to maintain a high level of executive vigilance (e.g., the Sustained Attention to Response Task, SART), which aligns with the real-world demands of multi-tasking and long-term vigilance. Our core finding, that alpha-1 power can serve as an EEG biomarker to guide the timing of transcranial direct current stimulation (tDCS) over the right posterior parietal cortex (rPPC), offers a new non-pharmacological intervention pathway for clinical populations with sustained attention deficits. These populations generally exhibit weak vigilance maintenance and a decline in performance during long-duration tasks, and traditional interventions are often limited by either the lack of precise temporal guidance or the risk of side effects. For example, patients with traumatic brain injury (TBI) demonstrate significantly poorer vigilance during target-monitoring tasks, which impacts their ability to live independently and their rehabilitation progress [73]. Similarly, children with attention-deficit/hyperactivity disorder (ADHD) exhibit a typical inattention pattern when performing attention network tasks (ANT), which negatively affects their academic and social functioning [74].

Although recent studies have confirmed the modulation effects of tDCS on certain cognitive functions, there are key differences between existing protocols and our approach. A meta-analysis of tDCS in ADHD children showed only small-to-medium effects on inhibitory control and working memory, with only one study using an “online stimulation” protocol (stimulus concurrent with task execution), while others used offline stimulation (stimulus applied before the task). The advantage of our study lies in filling this gap, providing precise temporal guidance through EEG biomarkers, which is crucial for clinical translation [74].

From a neurophysiological perspective, this study further validates the clinical potential of alpha-1 power as a marker for vigilance decline and reveals the specificity of tDCS modulation. During long-duration tasks, the brain typically shows an increase in alpha power, which is commonly associated with attention decline and mental fatigue. By applying tDCS to the right posterior parietal cortex (PPC), we were able to significantly suppress the trend of alpha power increase in the parietal region [75]. Although we did not find a direct link between alpha power changes and behavioral performance, these neurophysiological effects provide key insights into how tDCS may improve attention and offer a foundation for optimizing stimulation parameters to enhance intervention precision.

Although we did not directly test the effects in clinical populations, our findings lay the groundwork for future translational research. On one hand, the “visual modified Bakan task” used in this study can be simplified into a commonly used attention assessment paradigm, making it easier to integrate into clinical evaluation processes. On the other hand, monitoring alpha-1 power requires no complex equipment, making it suitable for application in rehabilitation centers or school settings. Future research could further explore personalized intervention thresholds for alpha-1 power in different populations and whether long-term repeated stimulation can sustain vigilance improvements. These directions, grounded in the “timing-effect” relationship established in this study, will ultimately advance precision neuromodulation in clinical interventions for attention disorders.

## 5. Limitations and Future Directions

The EEG analysis in this study primarily relied on inter-group comparisons with the blank control group. Although this design helps reveal the overall effects of tDCS, it also has several key limitations that warrant further investigation in future research. First, tDCS may introduce EEG artifacts related to the electrical current, such as baseline shifts and high-frequency noise. Although we minimized the impact of these artifacts through offline preprocessing (e.g., using Independent Component Analysis (ICA) to remove stimulation-related components and applying a 0.5–30 Hz band-pass filter) [13], potential confounding effects on EEG metrics, such as alpha-1 power, may still occur. Second, during the task, participants may experience practice effects, and vigilance naturally declines over time, which may overlap with the effects of tDCS intervention. Relying solely on inter-group comparisons makes it difficult to fully disentangle the influence of these non-specific factors, thus affecting the precise understanding of the intervention’s effects [57].

To gain a more comprehensive interpretation of the results, future studies could further explore intra-group pre-stimulation and post-stimulation changes. For example, in the late-stimulated group, comparisons could be made between the “pre-stimulation window (e.g., stage 2, before stimulation)” and the “post-stimulation window (e.g., stage 4, after stimulation)” in terms of alpha-1 power and behavioral metrics (e.g., hit rate, reaction time). This intra-group dynamic comparison would more directly reflect the immediate effects of stimulation and reduce the interference of individual differences between groups, thus allowing for a more accurate quantification of causal effects. Furthermore, finer time segmentation (e.g., every 2 min as a sub-phase) could be incorporated to more rigorously analyze the specific effects of stimulation.

## 6. Conclusions

This study investigated the influence of tDCS timing on the efficacy of vigilance enhancement through a controlled experimental design. The principal finding is that the timing of stimulation during the vigilance decrement process significantly modulates the effectiveness of tDCS intervention. In terms of objective vigilance performance, the tDCS intervention administered at a stimulation time point guided by mean alpha-1 power exhibited significantly greater efficacy when applied during the later stage of the task, as compared to tDCS delivered during the early stage. The present findings underscore the differential effects of tDCS intervention administered at varying levels of vigilance, highlighting the potential of exploring candidate stimulation time windows to enhance the efficacy of tDCS in mitigating vigilance decrement. Moreover, the use of alpha-1 power as an EEG-based indicator of vigilance in this study provides further empirical validation of the established association between alpha-1 activity and vigilance. It provides support for the broader application of alpha-1 power as an index for vigilance assessment.

## Figures and Tables

**Table 1:** Demographics. A summary of participant characteristics for the present study.

	Participants (n = 117)
Age: mean years ± SD (range)	20.56 ± 1.48 (18–25)
Gender	
Female	58
Male	59
Education	
Bachelor’s degree	64
Postgraduate degree	53
Handedness	
Right-handed	117
Left-handed	0
Mixed-handed	0
Visual Acuity	
Normal or Corrected-to-Normal Vision	117
Color Vision	Normal, n = 117

**Table 2:** Item Visual Analogue Mood Scale (VAMS).

Factors	Numbers	Scales	
vigilance	1	Alert	Drowsy
	2	Attentive	Dreamy
	3	Energetic	Lethargic
	4	Clear-headed	Muzzy
	5	Well-coordinated	Clumsy
	6	Quick-witted	Mentally slow
	7	Strong	Feeble
	8	Interested	Bored
	9	Proficient	Incompetent
Contentedness	10	Happy	Sad
	11	Amicable	Antagonistic
	12	Tranquil	Troubled
	13	Contented	Discontented
	14	Gregarious	Withdrawn
Calmness	15	Calm	Excited
	16	Relaxed	Tense

**Table 3:** Comparison between different groups of alpha-1 power.

Group (alpha-1 Power)	M	SD	t	*p*	Cohen’s d
Blank Control Group Stage III	20.3	2.19	−0.135	0.893	−0.33
Early-stimulated Group Stage III	20.22	2.88			
Blank Control Group Stage V	20.40	3.11	−3.24	0.002 **	−0.797
Late-stimulated Group Stage V	18.39	1.75			
Stage III differential	0.85	3.55	2.17	0.034 *	0.533
Stage V differential	2.01	3.66			

Note: * *p* < 0.05, ** *p* < 0.01.

## Data Availability

The data supporting the findings of this study are not publicly available due to ethical and privacy restrictions imposed by the Academic Ethics Committee of Guangdong University of Technology. However, anonymized summary data may be made available upon reasonable request to the corresponding author.

## Abbreviations

The following abbreviations are used in this manuscript:

TDCS	Transcranial direct current stimulation
EEG	electroencephalographic
